# Evaluation of a prerequisite course of histology implementation for Chinese students of eight-year medical programme: a mixed quantitative survey

**DOI:** 10.1186/s12909-022-03531-3

**Published:** 2022-07-01

**Authors:** Yan Ruan, Junlei Zhang, Qiyan Cai, Jiali Wang, Gaoke Liu, Yunlai Liu, Feng Mei, Jianqin Niu, Lan Xiao, Yanping Tian, Hongli Li

**Affiliations:** 1 Department of Histology and Embryology, College of Basic Medical Science, Army Medical University, Shapingba District, 30# Gaotanyan St, Chongqing, 400038 China; 2 Experimental Center of Basic Medicine, College of Basic Medical Science, Army Medical University, Chongqing, 400038 China

**Keywords:** Prerequisite course, Histology, 8-year medical programme, Active learning

## Abstract

**Background:**

Due to insufficient basic medical knowledge and inappropriate learning strategies, students of 8-year medical programme encountered many obstacles in the initial stage of basic medicine learning. This study was to determine whether a prerequisite course can improve basic medicine learning performance and adjust learning strategies to adapt to basic medicine learning.

**Methods:**

A prerequisite course of histology was constructed by a two-round modified Delphi study. Seventy-four students of 8-year medical programme were subjected to two groups: the prerequisite course group (PC group) and non-prerequisite course group (NPC group). The PC group take part in the prerequisite course by student-centred blended learning approach but NPC group not. The PC and NPC group underwent requisite histology teaching activities after prerequisite course. Examination of the prerequisite course and requisite histology course were carried out. Effect of the prerequisite course was evaluated by an empirical method using a questionnaire-based approach.

**Results:**

The results of examinations showed students' scores of the PC group were significantly higher than those of students of NPC group in both prerequisite course and requisite histology examinations (*P* < 0.05). The results of questionnaires showed that students were satisfied with the prerequisite course, which was beneficial for uptake in medical knowledge, cultivation of clinical thinking and scientific research ability and adaptation in learning strategies (*P* < 0.01). Furthermore, our prerequisite course is conducive to subsequent courses learning, especially for pathology (*P* < 0.01).

**Conclusion:**

Our prerequisite course could effectively supplement knowledge of basic medicine, improve clinical thinking and scientific research ability and adapt their learning strategies. These findings suggest that the prerequisite course is useful and should be introduced in medical curriculum reform at the early stages of basic medical training.

**Supplementary Information:**

The online version contains supplementary material available at 10.1186/s12909-022-03531-3.

## Background

Accompanying economic development and growth, it is a challenge to provide high quality medical services in China. A massive reform in medical education has already embarked on [1]. In 2001, program for the reform and development of medical education was approval to pilot an 8-year medical education programme to accelerate the training of high-quality clinicians [2]. Five-year programme is the primary pathway for most medical clinicians in China. In contrast, 8-year programme are designed to train high-quality clinician-scientists with rich clinical skills and scientific research ability [2–4]. Students of 8-year medical programme, who are awarded the doctor degree, are required to learn more in a short period of time because of the reduced duration of the training [5].

Eight-year medical programme is designed a long learning process, which includes premedical education in the early stage, basic medicine (pre-clinical) education in the middle stage, and clinical and research training in the later stage [5, 6]. Premedical courses include mathematics, physics, chemistry and so on. Students have appropriate learning methods and rich knowledge accumulation in high school to learn them [6]. However, basic medicine learning is different from that of premedical education, which requires abundant biological knowledge and appropriate learning methods. Medical students in North American, who have completed 3–4 years of undergraduate education, have extensive biomedical knowledge for clinical medicine training. However, similar to many Europe countries and Japan, Chinese students go to medical school directly from high school [7]. This situation leads to the lack of biological knowledge, which resulted in many obstacles to basic medicine learning for students of 8-year medical programme. At the basic medicine education stage, students need to learn a lot of knowledge. The student-centred approach was employed to advance student’s learning basic knowledge and train learning skills in college [8]. Unfortunately, students in high school are accustomed to a traditional, teacher-centered education, which lead to difficulties in active learning [9, 10]. It is more difficult for students of 8-year medical programme to change learning strategies at the initial stage of basic medicine.

Moreover, as clinical scientists, the 8-year medical programme students must develop clinical thinking and scientific research ability as early as possible, which should start training at the initial stage of basic medicine [11]. Histology is a core basic medicine component. Teaching implementation of histology is carried out at the initial phase of basic medical curricula, which is also the beginning of clinical thinking and scientific research training [12, 13]. Histology learning requires abundant biomedical knowledge and an appropriate study strategy for 8-year medical students [14]. A prerequisite course provides an opportunity to present students with a given knowledge or learning skills used. Forester et al. reported that the students taken prerequisite histology or anatomy course earned a significantly higher course grade in histology or anatomy course [15, 16].

In order to supplement biological knowledge, adjust learning strategies and cultivate clinical thinking and research ability in the initial stage of basic medicine, a prerequisite course of histology was conducted based on the cultivation goal of 8-year medical programme. Through the implementation of prerequisite course, the students learned more biological knowledge, adjusted study strategies from passive teacher-centred learning to active student-centred learning, and improved clinical thinking and scientific research capacity. These findings suggest that our prerequisite course is useful and should be introduced in medical curriculum reform at the early stages of basic medical training.

## Methods

### Participant sampling

The participating students were 74 undergraduates of 8-year medical programme at Army Medical University (Chongqing China) and divided into two groups: prerequisite course group (PC group, 39 persons) and non-prerequisite course group (NPC group, 35 persons). Admission data (age, gender, and a pretest score) were collected to establish baseline characteristics between the two groups. All students were tested for their histology knowledge before prerequisite course activities. Details of the paper are shown in supplementary materials (Additional file 1). Table 1 showed the basic characteristics of the two groups.

**Table 1:** Descriptive statistics of participant characteristics

Variables	PC group Number (%)	NPC group Number (%)	P
**Number**	39 (100%)	35 (100%)	
**Sex (M/F)**			0.45
Male	31 (79.5%)	29 (82.9%)	
Female	8 (21.5%)	6 (17.1%)	
**Mean age (years)**	21.14 ± 0.87	20.56 ± 0.95	0.14

### The construction of prerequisite course

A two-round modified Delphi study on teaching contents of prerequisite course was performed by teachers of cell biology, teachers of anatomy, and the faculty of department of Histology and Embryology in our university [17–19]. The final main content of prerequisite course and teaching model are as follows (Table 2).

- 1. Learning resources. The relevant textbooks and materials including relevant scientific research progress in Chinese and English were prepared. Resources such as online examination, online microscopy and clinical cases bank (An example in additional file 2) were constructed. Massive open online courses (MOOCs) were also used (https://www.icourse163.org, https://www.cnmooc.org or https://www.pmphmooc.com) [20]. Nineteen prerequisite concepts were designed.
- 2. Learning strategies. Teachers systematically introduce the strategies and learning methods of histology. Active learning patterns, student-centred learning, were established to replace the previous passive learning model, teacher-centred learning.
- 3. Blended learning. 39 students were randomly divided into 13 smaller groups, each composed of 3 participants. Through self-study, students master the relevant basic knowledge. Team members presented their results and held discussions with the other groups. Teachers provided guidance throughout the whole process by email, wechat, etc. and also summarized key and difficult points of the course by face-to-face.

**Table 2:** The concepts of prerequisite course of histology learning

Prerequisite topics	Detailed objectives
Learning contents	Cell biology: Cell membrane; mitochondrion; endoplasmic reticulum; ribosome; lysosome; cytoskeleton; nuclear
	Basic tissues: Epithelial tissue; connective tissue; muscle tissue; nervous tissue
	Systematic anatomy and organ histology: Nervous system; circulatory system; immune system; endocrine system; digestive system; respiratory system; urinary system; reproductive system
Learning strategies and resources	Learning strategies: the strategies and learning methods of histology
	Resources: the relevant textbooks and materials including relevant research progress in Chinese and English, massive open online courses (MOOCs), online examination, online microscopy and clinical cases bank
Teaching model	Blended learning: students grasp the relevant basic knowledge through self-study. Teachers provided guidance and summarized key points of the course face-to-face

### Teaching methods

#### PC group

The prerequisite course is carried out in semester before requisite histology course based on the implementation plan. After the prerequisite course, requisite histology course was undertaken by traditional teaching methods.

#### NPC group

The 35 students did not participate in prerequisite course, but underwent requisite histology teaching activities directly, provided by the same staff as with the PC group.

### Evaluation methods

Three approaches were used to evaluate effect of prerequisite course.

- 1. Pre-test assessment. A pre-test was carried out to test the grasp of basic knowledge in both the PC and NPC groups before and after prerequisite course.
- 2. Histology examination. Students in both PC and NPC groups took a final examination after finishing requisite histology teaching activities. The scoring staff were blinded to the identity of the students and their assigned group.
- 3. Questionnaire survey. After the end of the requisite course, the satisfaction with prerequisite course was evaluated by an empirical method using a questionnaire-based approach. Part of the survey was conducted two years after the prerequisite course. The scopes of the questionnaire included histology learning, subsequent courses (pathology) learning, clinical thinking and scientific research ability training and active learning. Each item of questionnaire was rated on a 4-point Likert scale described as, 1 = “completely insufficient for my opinion” to, 4 = “completely sufficient for my opinion”.

### Statistical analyses

The data from the students’ evaluation ratings was summarized using descriptive statistics (means, standard deviation (SD), and response rates). Statistical analysis was conducted using SPSS 20.0 software for Windows (SPSS Inc., Chicago, IL, USA). Data are presented as means ± SD. Statistical analysis between the groups was evaluated using t tests and analysis of variance (ANOVA). We used a *P*-value < 0.05 as the standard for statistical significance and a *P*-value < 0.01 as highly significant.

## Results

### Participation of PC and NPC groups

**Fig. 1:**
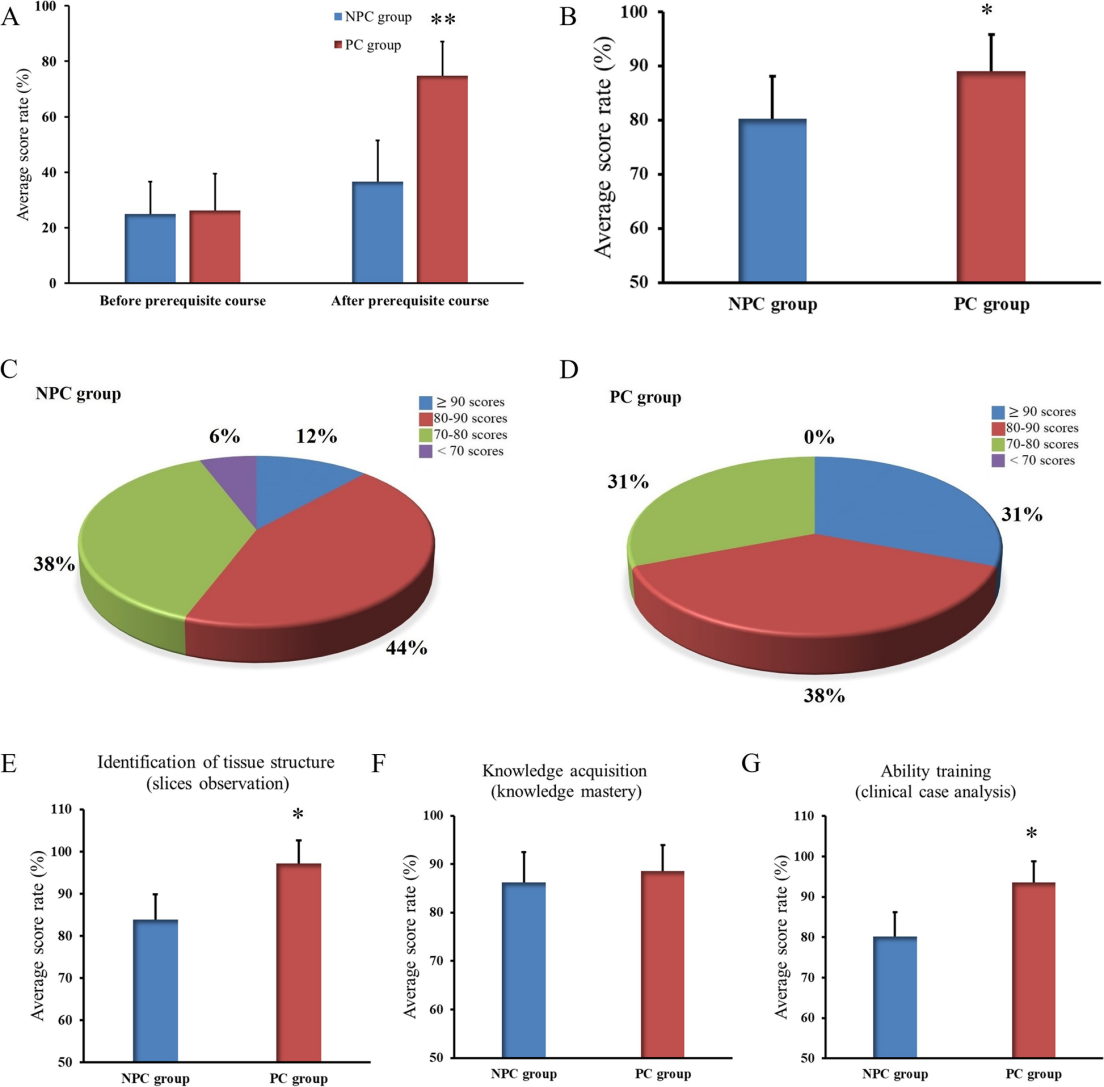
Results of the Examinations. **A** Average score rate of pre-test before and after prerequisite course. **B** Average score rate of final histology course. **C** Demographic characteristics of exam results of final histology course in NPC group. **D** Demographic characteristics of exam results of final histology course in PC group. **E** Average score rate of identification of tissue structure (slices observation). **F** Average score rate of knowledge acquisition (knowledge mastery). **G** Average score rate of ability training (clinical case analysis).*P < 0.05, **P < 0.01

The 74 participating students were divided into two groups: PC group (39 persons) and NPC group (35 persons). As evident in Table 1, there was no significant difference between the groups in terms of numbers, sex and age (*P* > 0.05). The results of a pre-test was also not different between the two groups before prerequisite course (Fig. 1A, *P* > 0.05). All the students took the final examination of histology and completed the questionnaire in this study.

### Examination results of PC and NPC groups

Students mastered sufficient biological knowledge in prerequisite course.

To determine the impact of the prerequisite course on knowledge acquisition, students in both groups completed the test before (pre) and after (post) the prerequisite course and the scores in the pre-test and post-test were analyzed (Fig. 1A). We found no difference in the test scores between PC group and NPC group before the course. However, the scores of the PC group after the course (mean ± SD, 74.8 ± 14.9) were significant higher than those of the NPC group (36.5 ± 12.3; Fig. 1A; *P* < 0.01).

The prerequisite course increased scores of histology examination.

The final exam scores of requisite histology course were also statistically analyzed. Results showed that students of PC group scored significantly higher than that of students not participated in the prerequisite course (Fig. 1B; *P* < 0.05). Further analysis showed the percentage of high scores (> 80) of the PC group was significantly higher than that of the NPC group (Fig. 1C, D). Higher achieving students in the PC group scored significantly higher on identification of tissue structure (slices observation) and ability training (clinical case analysis) than those in the NPC group (Fig. 1E, and G, *P* < 0.05). There was no significant difference in knowledge acquisition (knowledge mastery) between the two groups (Fig. 1F, *P* > 0.05).

### Analysis of questionnaire of PC and NPC groups

The prerequisite course was beneficial to histology and subsequent courses learning.

The students were asked to complete an anonymous questionnaire about the prerequisite course after the requisite course. The evaluation results showed that students had a high degree of satisfaction with the effect of our prerequisite course on histology learning. 97.5% of the students agreed that the prerequisite course was helpful for histology learning (*P* < 0.01). The prerequisite course was beneficial to master learning contents of histology. The students of PC group agreed that the prerequisite course helped them to observe the H&E-stained sections and analyze clinical cases efficiently (Table 3). The prerequisite course is also conducive to learning of other courses. 92.4% of students in PC group stated that the PC was beneficial to pathology study (*P* < 0.05). The study strategies of our prerequisite course promote efficiency of pathology learning (Table 4).

**Table 3:** Questionnaire results about effect of prerequisite course on histology learning in the PC and NPC groups

Items	PC group	NPC group	t	P
Helpful for histology learning	3.56 ± 0.09	3.09 ± 0.15	2.82	< 0.01
Increased interest in histology learning	3.39 ± 0.10	3.05 ± 0.13	2.01	0.04
Master learning strategies of histology	3.23 ± 0.12	2.66 ± 0.15	3.06	< 0.01
Help us to improve our learning efficiency	3.31 ± 0.11	2.66 ± 0.15	3.45	< 0.01
I know the goal of histology learning	3.05 ± 0.13	2.49 ± 0.14	2.96	< 0.01
Master many basic knowledge of histology	3.56 ± 0.10	2.46 ± 0.12	6.89	< 0.01
Helpful for slices observation	3.46 ± 0.11	2.54 ± 0.14	5.27	< 0.01
Helpful for clinical case analysis	3.39 ± 0.12	2.40 ± 0.12	5.70	< 0.01
Master professional English of histology	3.34 ± 0.15	2.54 ± 0.14	5.24	< 0.01

**Table 4:** Questionnaire results about effect of prerequisite course on later courses (Pathology) learning in the PC and NPC groups

Items	PC group	NPC group	t	P
Helpful for pathology learning	3.39 ± 0.10	3.00 ± 0.15	2.18	0.03
Master learning strategies of pathology	3.18 ± 0.12	2.60 ± 0.14	3.11	< 0.01
Improve learning efficiency of pathology	3.23 ± 0.11	2.71 ± 0.15	2.77	< 0.01
Helpful for pathological slices observation	3.39 ± 0.11	2.63 ± 0.14	4.18	< 0.01
Helpful for clinical pathological case analysis	3.21 ± 0.13	2.46 ± 0.13	4.06	< 0.01
Conducive to learn other courses	3.31 ± 0.11	2.31 ± 0.13	5.88	< 0.01

The prerequisite course improved students’ clinical thinking and scientific research ability.

Our prerequisite course also provided clinical thinking and scientific research training (Table 4 and 5). The results of questionnaire showed that knowledge and skills they learned from PC course is helpful for the observation of pathological tissue slices and the analysis of clinical pathological case (*P* < 0.01). The morphological observation and analysis on slices improved their scientific research ability and are conducive to subsequent scientific research tasks (*P* < 0.01). 84.7% of students of PC group agreed that English materials increased their vocabulary and improved writing ability with professional English (*P* < 0.01).

**Table 5:** Questionnaire results about effect of prerequisite course on scientific research ability training in the PC and NPC groups

Items	PC group	NPC group	t	P
Master professional English of histology	3.28 ± 0.13	2.83 ± 0.13	2.47	0.02
Increase my professional English vocabulary	3.31 ± 0.12	2.66 ± 0.14	3.57	< 0.01
Improve my English writing ability	3.23 ± 0.11	2.54 ± 0.14	3.89	< 0.01
Helpful for slices observation	3.41 ± 0.11	3.06 ± 0.12	2.23	0.03
Helpful for morphological analysis	3.18 ± 0.14	2.57 ± 0.12	3.26	< 0.01
Improve my clinical analysis ability	3.31 ± 0.12	2.91 ± 0.12	2.36	0.02
Improve my scientific research ability	3.31 ± 0.14	2.74 ± 0.13	2.94	< 0.01
Expand my academic field	3.38 ± 0.12	2.66 ± 0.14	4.04	< 0.01
Conducive to subsequent scientific research tasks	3.37 ± 0.12	2.63 ± 0.13	4.26	< 0.01
I would recommend this course to my peers	3.44 ± 0.09	2.54 ± 0.17	4.78	< 0.01

The prerequisite course adjusted students’ learning strategies.

The blended approach offers opportunities for student-centered active learning. Table 6 summarizes students’ perceptions of the student-centred blended learning. In the feedback analysis the majority of students considered active learning methods useful for histology learning. 87.2% of the students were satisfied (3 and 4 on the four-point scale) with the blended learning (*P* < 0.01). The students regarded blended learning as an innovative learning method and agreed that it helps them to learn more efficiently and enhance integration with prerequisite content.

**Table 6:** Questionnaire results about learning strategies in the PC and NPC groups

Items	PC group	NPC group	t	P
I like face- to-face teaching	2.80 ± 0.15	3.54 ± 0.11	3.89	< 0.01
I like e-learning	2.92 ± 0.15	2.80 ± 0.16	0.57	0.57
I like blended learning	3.46 ± 0.12	2.66 ± 0.14	4.55	< 0.01
E-learning is a replacement for face- to-face teaching	1.80 ± 0.14	2.40 ± 0.15	2.98	< 0.01
Teachers play a better role for guiding	3.56 ± 0.9	3.63 ± 0.08	0.53	0.59
Blended learning helps me to learn more efficiently	3.41 ± 0.11	2.54 ± 0.15	4.76	< 0.01
Blended learning enables me to learn in a pleasant atmosphere	3.30 ± 0.13	2.54 ± 0.11	4.38	< 0.01
Blended learning enables me to integrate prerequisite content	3.70 ± 0.11	2.23 ± 0.14	8.42	< 0.01
Blended learning enables me to study actively	3.72 ± 0.09	2.66 ± 0.14	6.65	< 0.01
I was generally satisfied with the blended learning	3.51 ± 0.10	2.46 ± 0.14	6.21	< 0.01
I wish more blended learning courses available	3.44 ± 0.10	3.00 ± 0.11	3.02	< 0.01

## Discussion

Eight-year medical programme in China are designed to train high-quality clinician-scientists. But students are enrolled from high school, which makes it difficult to learn basic medicine courses due to insufficient biological knowledge and inappropriate learning methods. In this project, the prerequisite course provides a good opportunity to help students supplement basic medical knowledge and adjust their learning strategies and cultivate clinical thinking and research ability in the initial stage of basic medicine.

Supplement of basic medical knowledge to support basic medicine courses learning.

Histology is a visually oriented, foundational anatomical sciences subject, which is learned at early stage of basic medical curricula. There are many difficulties for undergraduate students in learning histology, such as understanding of terminologies and relationships between tissues and related diseases [21]. Sufficient biological knowledge is required for histology learning, which learned at high school not support histology learning. The prerequisite course may provide more biology knowledge [16]. We provided knowledge of systemic anatomy, cell biology and histology, as well as numbers of clinical cases and online resources. The students acquired sufficient basic medical knowledge, which enhanced student learning in later requisite histology course. The results showed our prerequisite course promoted students to master sufficient knowledge, which is useful to support histology learning. As the basis for the pathology, the mastery of histological knowledge affect pathology learning. Markus et al. reported that histological knowledge is a predictor of medical students’ performance in diagnostic pathology [22]. The knowledge learned in our course supports pathology learning. Therefore, our prerequisite course is beneficial for basic medicine courses learning.

Improving clinical thinking and scientific research ability to train high-quality clinician-scientists.

The goal of the 8-year medical programme is to train high-quality clinician-scientists, who have not only rich clinical thinking but also multiple scientific research skills [3]. Although the clinical thinking and research ability of students in 8-year medical programs is a pivotal quality, it remains unclear of cultivation measures and methods [11]. Therefore, we added the content of cultivating students' clinical thinking and scientific research ability. Our prerequisite course provides clinical cases and professional English materials, including scientific research papers. Clinical thinking is developed by analyzing clinical cases. Sufficient clinical cases knowledge lay a good foundation for students to develop clinical thinking, which is supported by our findings and other reports [12]. We also provide section observation and morphological analysis training, which are important methods of scientific research. Professional English learning helps students to read and write scientific research papers. Therefore, our course not only provide rich basic knowledge but also cultivate clinical thinking and scientific research ability for students of 8-year medical programme.

Changing learning strategies to improve learning efficiency.

Appropriate learning strategies accelerate students to master relevant knowledge and skills. High school education in China has been characterized by a style of teacher-centred didactic teaching. Faculty transmit knowledge too much in a too short time, while students are expected to be passive recipients of knowledge, which reduces time to develop abilities such as critical thinking, problem solving, and clinical decision making [23]. The student centred approach, focusing on the learners' learning, rather than the teacher's teaching, is an active learning strategy, which is potential to advance student not only learning basic knowledge but also training critical thinking and learning skills [24, 25]. Active learning strategies commonly include problem-based learning, flipped classroom and blended learning [26–28]. Blended learning used in our prerequisite course changes a teacher-centred, content driven approach to a student-centred, process-driven approach, which is expected to generate independent, active, and autonomous learners [28, 29]. Students use resources teachers provide and online resources to learn actively. Teachers help students solve them face-to-face when students are confronted with problems. Most students find this learning strategy promotes their learning efficiency. The results suggest that students established student centred learning methods suitable for basic medicine courses.

## Limitations

A general limitation of this study was that the number of participants was relatively small, and therefore not provided more information about the prerequisite course. Increase the number of participating students and prolongs of the implementation time is to evaluate the effect of our prerequisite course in the future. Moreover, minority participants were negative about the prerequisite course. Our further analysis found that it was more difficult for those students to adapt to a student-centered teaching approach in a short time [30]. It took longer time for these students to change their learning strategies.

## Conclusions

Our findings indicate that our prerequisite course, a specific course for 8-year medical programme undergraduate, is a powerful educational strategy, which helps students not only increasing basic medical knowledge, improving clinical thinking and scientific research ability but also adjusting their learning strategies. Our prerequisite course is useful for curriculum development and resources support to objectives of 8-year medical programme. These findings suggest that our prerequisite course should be incorporated into the medicine curriculum reform at the initial stage of basic medicine.

## Supplementary Information

**Additional file 1:** (DOC 70 kb)

**Additional file 2:** (DOCX 13 kb)

## Data Availability

No data are shared. If anyone need our data and materials, we are very pleased to provide (contact Prof. Hongli Li (lihlimm@163.com) or Yanping Tian (tianyp1981@163.com) who can provide data).

## References

[1] Huang L, Cai QL, Cheng LM, Kosik R, Mandell G, Wang SJ, Xu GT, Fan AP (2014). Analysis of Curricular Reform Practices at Chinese Medical Schools. Teach Learn Med.

[2] Wang Z, Yin Z, Wei YB, Liu LF, Yang JR (2015). The expansion of 8-year medical training programs in China: a status report. Med Educ Online.

[3] Baumal R, Benbassat J, Van JA (2014). Reflections on the current and future roles of clinician-scientists. The Israel Medical Association journal : IMAJ.

[4] Brown NJ (2018). Developing Physician-Scientists. Circ Res.

[5] Ren X, Yin J, Wang B, Roy Schwarz M (2008). A descriptive analysis of medical education in China. Med Teach.

[6] Zhang Q, Lee L, Gruppen LD, Ba DN (2013). Medical education: Changes and perspectives. Med Teach.

[7] Zavlin D, Jubbal KT, Noe JG, Gansbacher B (2017). A comparison of medical education in Germany and the United States: from applying to medical school to the beginnings of residency. Ger Med Sci.

[8] Hurney CA (2012). Learner-centered teaching in nonmajors introductory biology: the impact of giving students choices. J Microbiol Biol Educ.

[9] Wang W (2021). Medical education in china: progress in the past 70 years and a vision for the future. BMC Med Educ.

[10] Jian W, Qi Z, Xin D (2011). Preliminary research in the application of integrated learning and teacher-centredness in undergraduate education in China. Med Teach.

[11] Wan M, Liu S, Zhu J, Xiao S, Yuan L, Lei X, Lei H, Shi X, You W, Ruan G (2022). Challenges of senior 8-year-program medical students' scientific research in China: A multicenter questionnaire-based study. Medicine.

[12] Zaletel I, Maric G, Gazibara T, Rakocevic J, LabudovicBorovic M, Puskas N, Bajcetic M (2016). Relevance and attitudes toward histology and embryology course through the eyes of freshmen and senior medical students: Experience from Serbia. Ann Anat.

[13] Cheng X, Chan LK, Li H, Yang XS (2020). Histology and Embryology Education in China: The Current Situation and Changes Over the Past 20 Years. Anat Sci Educ.

[14] Selvig D, Holaday LW, Purkiss J, Hortsch M (2015). Correlating students’ educational background, study habits, and resource usage with learning success in medical histology. Anat Sci Educ.

[15] Forester JP, McWhorter DL, Cole MS (2002). The relationship between premedical coursework in gross anatomy and histology and medical school performance in gross anatomy and histology. Clin Anat.

[16] Sato BK, Lee AK, Alam U, Dang JV, Dacanay SJ, Morgado P, Pirino G, Brunner JE, Castillo LA, Chan VW (2017). What’s in a Prerequisite? A Mixed-Methods Approach to Identifying the Impact of a Prerequisite Course. CBE Life Sci Educ.

[17] Swamy M, Venkatachalam S, McLachlan J (2014). A Delphi consensus study to identify current clinically most valuable orthopaedic anatomy components for teaching medical students. BMC Med Educ.

[18] Kelsch MP, Sylvester RK: The Effect of Prerequisite Pharmacodynamics Course Timing on Student Performance in Pharmacotherapy Courses. Am J Pharm Educ 2016, 80(6). 10.5688/ajpe80699 PMC5023991 27667836

[19] Guo Y, Li E (2016). Collaborative Testing in Practical Laboratories: An Effective Teaching-Learning Method in Histology. J Vet Med Educ.

[20] Gong Z (2018). The development of medical MOOCs in China: current situation and challenges. Med Educ Online.

[21] Garcia M, Victory N, Navarro-Sempere A, Segovia Y (2019). Students' Views on Difficulties in Learning Histology. Anat Sci Educ.

[22] Nivala M, Lehtinen E, Helle L, Kronqvist P, Paranko J, Saljo R (2013). Histological Knowledge as a Predictor of Medical Students' Performance in Diagnostic Pathology. Anat Sci Educ.

[23] Matsuyama Y, Nakaya M, Okazaki H, Lebowitz AJ, Leppink J, van der Vleuten C (2019). Does changing from a teacher-centered to a learner-centered context promote self-regulated learning: a qualitative study in a Japanese undergraduate setting. BMC Med Educ.

[24] Jacobs JC, van Luijk SJ, Galindo-Garre F, Muijtjens AM, van der Vleuten CP, Croiset G, Scheele F (2014). Five teacher profiles in student-centred curricula based on their conceptions of learning and teaching. BMC Med Educ.

[25] Kar SS, Premarajan KC, Subitha L, Archana R, Iswarya S, Sujiv A (2014). Student-centred learning in Community Medicine: An experience from Jawaharlal Institute of Postgraduate Medical Education and Research. Puducherry Natl Med J India.

[26] Jin J, Bridges SM (2014). Educational technologies in problem-based learning in health sciences education: a systematic review. J Med Internet Res.

[27] Hew KF, Lo CK (2018). Flipped classroom improves student learning in health professions education: a meta-analysis. BMC Med Educ.

[28] Westerlaken M, Christiaans-Dingelhoff I, Filius RM, de Vries B, de Bruijne M, van Dam M (2019). Blended learning for postgraduates; an interactive experience. BMC Med Educ.

[29] Morton CE, Saleh SN, Smith SF, Hemani A, Ameen A, Bennie TD, Toro-Troconis M (2016). Blended learning: how can we optimise undergraduate student engagement?. BMC Med Educ.

[30] Venkatesh S, Rao YK, Nagaraja H, Woolley T, Alele FO, Malau-Aduli BS (2020). Factors Influencing Medical Students' Experiences and Satisfaction with Blended Integrated E-Learning. Med Princ Pract.

