# Supplementary material for: Evaluation of a prerequisite course of histology implementation for Chinese students of eight-year medical programme: a mixed quantitative survey
Source: BMC Med Educ. 2022 Jul 1;22:514. doi: 10.1186/s12909-022-03531-3 (PMC9248162; doi:10.1186/s12909-022-03531-3)
Supplement: Supplementary file 1 — Additional file 1: (DOC 70 kb) [file 12909_2022_3531_MOESM1_ESM.doc]

**一、选择题。在每小题给出的A、B、C、D、E五个选项中，只有一项是最符合题目要求的。每题2.5分，共50分。**

1. HE staining (hematoxylin-eosin staining) is a common technique in histological techniques. The nucleus and ribosomes (核糖体) are stained blue with hematoxylin. What function of ribosomes? （ ）

A. Synthesis of proteins B. Synthesis of nucleic acid

C. Synthesis of fatty D. Synthesis of DNA

E. Synthesis of RNA

2. The cell junction specialized for cell-cell communication is called a（ ）

A. Intermediate junction (中间连接) B. Tight junction(紧密连接)

C. Gap junction (缝隙连接) D. Desmosome (桥粒)

E. Hemidesmosome (半桥粒)

3. Many people are allergic (过敏) to pollen. Which of the following cells is involved with allergy （ ）

A. Plasma cell B. Mast cell (肥大细胞)

C. Fibrocyte D. Fibroblast (成纤维细胞)

E. Gland cell

4. Strenuous exercise can lead to ligament (韧带) injury. Which of the following tissue is component of ligament. （ ）

A. Dense regular connective tissue B. Dense irregular connective tissue

C. Adipose tissue D. Reticular tissue (网状组织)

E. Ground substance

5. Severe bleeding may affect life. Which type of cells is associated with the allergic reaction and serves as an anticoagulant (抗凝血剂)? （ ）

A. Platelets B. Monocytes

C. Neutrophils (中性粒细胞) D. Basophils (嗜碱性粒细胞)

E. Eosinophils (嗜酸性粒细胞)

6．The main function of red cell is oxygen transport. Which of the following structures is associated with this function? （ ）

A.Nuclear protein B. Actin

C．Spectrin(血影蛋白) D．Hemoglobin (血红蛋白)

E. Albumin (白蛋白)

7. Parkinson's disease (PD) is a degenerative disorder of the central nervous system. The motor symptoms of Parkinson's disease result from the death of dopamine-generating cells in the substantia nigra, a region of the midbrain. Which of the following cell express dopamine (多巴胺)? （ ）

A. Neuron B. Oligodendrocytes (少突胶质细胞)

C. Astrocytes (星形胶质细胞) D. Microglia

E. Fibroblasts

8. Every day we see many beautiful pictures. The cell type that carries neural transmission from the retina (视网膜) to the brain is the（ ）

A. Ganglion cell (节细胞) B. Amacrine cell (无长突细胞)

C. Bipolar cell (双极细胞) D. Horizontal cell (水平细胞)

E. Interplexiform cell (网间细胞)

9. The immune system is an important component of the body protection systems, in which lymphoid nodule is an important lymphoid tissue. The lymphoid nodules are NOT present in the（ ）

A. Thymus (胸腺) B. Spleen

C. Lymph nodes D. Palatine tonsils (腭扁桃体)

E. Ileum (回肠)

10. AIDS is a serious infectious disease, HIV disrupts the body's immune cells. Which of the following cell is not immune cells

A. B cell B. T cell

C. Nature killer cell D. Chromaffin cells (嗜铬细胞瘤)

E. Antigen presenting cell

11. Aldosterone (醛固酮) is a steroid hormone which plays a central role in the regulation of blood pressure mainly by acting on the distal tubules and collecting ducts of the nephron, increasing reabsorption (重吸收) of ions and water in the kidney, to cause the conservation of sodium, secretion of potassium, increased water retention, and increased blood pressure. Aldosterone is secreted by the（ ）

A. Zona reticularis (网状带) B. Chromaffin cells

C. Chromophobe cells (嫌色细胞) D. Zona glomerulosa (球状带)

E. Pituicytes (垂体)

12. Cretinism (呆小症) is a condition of severely stunted physical and mental growth due to untreated congenital deficiency of ONE hormone. Which of the following hormones deficiency cause cretinism? （ ）

A. Thyroxine (甲状腺素) B. Calcitonin (降钙素)

C. Parathyroid hormone D. Aldosterone

E. Cortisol (皮质醇)

13. Excessive stomach acid digest the gastric mucosa (粘膜) named Gastric ulcer (胃溃疡), which is the clinical common disease, one of frequently-occurring diseases. Which of the following cell has gastric acid secretion capacity（ ）

A. Chief cell B. Parietal cell

C. Stem cell D. Muscle cells

E. None of above

14. More and more patients with diabetes appear in China. Which of the following hormone deficiency results in increase in blood glucose? （ ）

A. Glucagons (胰高血糖素) B. Somatostatin (生长抑素)

C. Insulin (胰岛素) D. Trypsinogen (胰蛋白酶原)

E.Lysozyme (溶菌酶)

15. The cells of which structure monitor the concentration of NaCl in the afferent arteriole of the glomerulus (肾小球)? （ ）

A. Proximal convoluted tubule (近曲小管) B. Distal convoluted tubule (远曲小管)

C. Mesangial cells (系膜细胞) D. Macula densa (致密斑)

E. Collecting ducts

16. The old men often have dysuria（排尿困难） because of which organ often found Concretions (凝固体) （ ）

A. Epididymis (附睾) B. Seminal vesicle (精囊腺)

C. Prostate (前列腺) D. Rete testis (睾丸网)

E. Ampullar region of the vas deferens (输精管壶腹部)

17. Liking a soap bubble collapse, alveolar (肺泡) in lungs is not collapse because it contains（ ）

A. Insulin B. Cilia

C. Lysozyme D. Somatostatin

E. Surfactant

18. The liver is the largest digestive gland in human body. Which of the following does hepatocyte secretes? （ ）

A. Bile B. Trypsin

C. Chymotrypsin (糜蛋白酶) D. Vitamin A

E. Pepsin (胃蛋白酶)

19. Birthing a healthy baby is the happiness of each family. The most easily pregnant (怀孕) time is in the female menstrual cycle? （ ）

A. 3-5 days B. 6-9 days

C. 12-16 days C. 20-23 days

E. 24-27 days

20. Long beard is a manifestation of the man secondary sexual characteristics. Which of the following hormone and are directly related to secondary sexual characteristics? （ ）

A. Follicle stimulating hormone (FSH) B. Luteinizing hormone (LH)

C. Aldosterone D. Androgen

E. The sugar cortical hormone (糖皮质激素)

**二、英文翻译题。将下列英文单词翻译成中文。每题1分，共50分。**

1. Histology 2. Embryology

3. Cilia 4. Microvilli

5. Endothelium 6. Mesothelium

7. Blood island 8. Isogenous group

9. Collagenous Fiber 10. Macrophage

11. Hyaline Cartilage 12. Haversian system

13. Sarcomere 14. Intercalated Disk

15. Synapse 16. Sinusoid

17. Myelin sheath 18. Ranvier Node

19. Blood Brain Barrier 20. Capillary

21. Internal elastic membrane 22. Recirculation of Lymphocyte

23. Mononuclear Phagocytic System 24. Thymic Corpuscle

25. Blood Thymus Barrier 26. Periarterial Lymphatic Sheath

27. Herring Body 28. Adrenaline

29. Intestinal Villus 30. Gastric pit

31. Pancreas Islet 32. Hepatic Lobule

33. Centroacinar cell 34. Portal Area

35. Alveolar sac 36. Blood -Air Barrier

37. Nephron 38. Podocyte

39. Filtration Barrier 40. Spiral organ

41. Seminiferous Tubule 42. Spermatogenesis

43. Spermiogenesis 44. Blood Testis Barriar

45. Follicle 46. Zona Pellucida

47. Corona Radiata 48. Ovulation

49. Corpus Luteum 50. Hypophyseal Portal System
