# Supplementary material for: Evaluation of a prerequisite course of histology implementation for Chinese students of eight-year medical programme: a mixed quantitative survey
Source: BMC Med Educ. 2022 Jul 1;22:514. doi: 10.1186/s12909-022-03531-3 (PMC9248162; doi:10.1186/s12909-022-03531-3)
Supplement: Supplementary file 2 — Additional file 2: (DOCX 13 kb) [file 12909_2022_3531_MOESM2_ESM.docx]

**Case:**

The patient is a 40-year-old male. Intermittent epigastric pain without obvious incentive began more than 10 years ago and became worse in the last 2 weeks. 6 hours ago, the patient experienced sudden abdominal distension with nausea and dizziness. He passed tarry starry stools twice and vomited coffee-like liquid once. Afterwards, he had palpitation, dizziness and cold sweat. **Physical examination**: Heart rate: 108 times/min; Blood pressure: 90/70mmHg. The face was pale with cold and clammy skin. Nothing abnormal was observed in reference to the heart and lungs. A mild tenderness was in the upper abdomen with no muscle tension or rebound tenderness. No abdominal mass was touched. Liver and spleen were not touched. Sign of ascites: negative. **Blood Routine test**: HB: 82g/L. WBC: 5.8×10^9^/L. Stool occult blood test: strongly positive. **Gastroscopy**: a 1.0cm×1.0 cm ulcer was observed near the posterior wall of the lesser curvature of the stomach.

**Question:**

(1) Describe the structure of gastric mucosa.

(2) Briefly describe the mechanisms of gastric mucosa self-protection.

(3) Tell the difference between an ulcer and erosion.

(4) Analyze the causes of gastric ulcer.
